# Comparative genomics and stable isotope analysis reveal the saprotrophic-pathogenic lifestyle of a neotropical fungus

**DOI:** 10.1128/mbio.01423-24

**Published:** 2024-07-16

**Authors:** Luiz Marcelo Ribeiro Tomé, Gabriel Quintanilha-Peixoto, Diogo Henrique Costa-Rezende, Carlos A. Salvador-Montoya, Domingos Cardoso, Daniel S. Araújo, Jorge Marcelo Freitas, Gabriela Bielefeld Nardoto, Genivaldo Alves-Silva, Elisandro Ricardo Drechsler-Santos, Aristóteles Góes-Neto

**Affiliations:** 1 Department of Microbiology, Molecular and Computational Biology of Fungi Laboratory, Instituto de Ciências Biológicas, Universidade Federal de Minas Gerais, Belo Horizonte, Brazil; 2 Departamento de Ciências Biológicas, Programa de Pós-graduação em Botânica, Universidade Estadual de Feira de Santana, Feira de Santana, Brazil; 3 MIND.Funga (Monitoring and Inventorying Neotropical Diversity of Fungi) - MICOLAB, Universidade Federal de Santa Catarina, Florianópolis, Brazil; 4 Fundación Miguel Lillo, Instituto Criptogámico-Sección Micología, San Miguel de Tucumán, Argentina; 5 Organización Juvenil “Hongos Perú”, Cusco, Santiago, Peru; 6 Instituto de Pesquisas Jardim Botânico do Rio de Janeiro (JBRJ), Rio de Janeiro, Brazil; 7 Instituto de Biologia, Universidade Federal da Bahia, Salvador, Brazil; 8 Program in Bioinformatics, Loyola University Chicago, Chicago, Illinois, USA; 9 Departamento de Ecologia, Universidade de Brasília, Brasília, Brazil; Institut Pasteur, Paris, France; Clark University, Worcester, Massachusetts, USA

**Keywords:** fungus-plant interactions, Hymenochaetaceae, Fabaceae, CAZy, C13/N15 stable isotopes

## Abstract

In terrestrial forested ecosystems, fungi may interact with trees in at least three distinct ways: (i) associated with roots as symbionts; (ii) as pathogens in roots, trunks, leaves, flowers, and fruits; or (iii) decomposing dead tree tissues on soil or even on dead tissues in living trees. Distinguishing the latter two nutrition modes is rather difficult in Hymenochaetaceae (Basidiomycota) species. Herein, we have used an integrative approach of comparative genomics, stable isotopes, host tree association, and bioclimatic data to investigate the lifestyle ecology of the scarcely known neotropical genus *Phellinotus*, focusing on the unique species *Phellinotus piptadeniae*. This species is strongly associated with living *Piptadenia gonoacantha* (Fabaceae) trees in the Atlantic Forest domain on a relatively high precipitation gradient. Phylogenomics resolved *P. piptadeniae* in a clade that also includes both plant pathogens and typical wood saprotrophs. Furthermore, both genome-predicted Carbohydrate-Active Enzymes (CAZy) and stable isotopes (δ^13^C and δ^15^N) revealed a rather flexible lifestyle for the species. Altogether, our findings suggest that *P. piptadeniae* has been undergoing a pathotrophic specialization in a particular tree species while maintaining all the metabolic repertoire of a wood saprothroph.

**IMPORTANCE:**

This is the first genomic description for *Phellinotus piptadeniae*. This basidiomycete is found across a broad range of climates and ecosystems in South America, including regions threatened by extensive agriculture. This fungus is also relevant considering its pathotrophic-saprotrophic association with *Piptadenia goanocantha*, which we began to understand with these new results that locate this species among biotrophic and necrotrophic fungi.

## INTRODUCTION

The ability to thrive in a living plant host is widespread in pathogenic and symbiotic fungi. A wide range of relationships can be described among the plant-fungal interactions. Fungi can be classified as biotrophic when they derive energy from living cells; necrotrophic, when energy is obtained from killed cells (they invade and kill plant tissue rapidly and then live saprotrophically on the dead remains); or hemibiotrophic, when they engage in an initial biotrophic phase followed by necrotrophy (1). Fungal pathogens attacking wood can be classified as heart-rot fungi, which are specialized in decomposing the heartwood of trees, with the colonization and decay process beginning while the host is alive. Otherwise, the decay may occur on sapwood, or the infection may advance from heartwood to sapwood (2).

The understanding of fungal symbiotic interactions and mechanisms involved in organic matter conversion are of high interest in both mycology and biotechnology. Using comparative genomics is a viable approach to this theme (3). Various studies show that different mechanisms could be related to the trophic mode of fungi, such as the presence and abundance of gene families related to the decaying of lignocellulosic material (4, 5). Furthermore, different nutrition modes in fungi can be correlated to different stable isotope signatures (such as carbon and nitrogen), allowing the distinguishing of whether nutrition is acquired from dead organic matter, or a living symbiont (6–9).

Hymenochaetales fungi exhibit a wide range of lifestyles, such as saprotrophic, mycorrhizic, bryophyllic, and plant pathotrophic (9, 10). A deep investigation of trophic modes in Hymenochaetales, based on stable isotope analyses, has revealed a greater diversity of trophic modes in this order than previously assumed. The species in Hymenochaetales can be broadly classified as saprotrophic (lignicolous species), or in other two groups of biotrophic taxa (although not necessarily parasitic), one composed mainly of ectomycorrhizae, and the second including ectomycorrhizae, saprotrophs, and briophyllous fungi (9). The family Hymenochaetaceae, the most representative in the order, encompasses several plant pathogens (11, 12). While these fungi frequently decompose dead portions of the trees (heart rot), cases in which some of these hymenochaetoid fungi attack the living and defense-active sapwood are also reported (2). Thus, it is debatable whether these pathogens would have isotope signatures closer to the saprotrophic, or one of the biotrophic clusters recovered by Korotkin et al. (9). In the same way, understanding whether the gene profile related to lignocellulose decay of those fungi tends to be more similar to non-pathogenic species (exclusively saprotrophs) or other trophic modes is also an open question.

The exclusively neotropical hymenochaetaceous species *Phellinotus piptadeniae* (Teixeira) Drechsler-Santos & Robledo has been described with an intriguing pathogenic mode (13–16). Recently, Salvador-Montoya et al. (2022) (14) refined the taxonomic concept of this species based on its morphology, distribution, and host association. Accordingly, the perennial basidiomata of *P. piptadeniae* are found on living trees of the legume species *Piptadenia gonoacantha* (Mart.) J.F. Macbr. (recurrent host), as well as other species of Fabaceae and Myrtaceae (14, 16). Based on the studies of Elias et al. (16) and Salvador-Montoya et al. (14), several collections of basidiomata of *P. piptadeniae* are recorded, mainly on living trees of *P. gonoacantha*, usually found on high branches of the trees, which eventually are aborted (break and fall off the tree), and rarely on the trunk, but also on dead branches of living trees.

To clarify plant pathogenic interactions in this ecologically interesting species, *Phellinotus piptadeniae*, its complete genome was newly sequenced, assembled, and annotated. We subjected this new genome to a phylogenomic and comparative genomics approach with other publicly available, high-quality assembled genomes of Hymenochaetales. Moreover, a molecular clock analysis was performed to evaluate whether the current taxonomic concept of the species comprises a hidden diversity. Finally, stable isotope signature (C13 and N15) of the species was accessed for the first time and compared with fungi from different trophic modes in Basidiomycota. The findings of our study reveal new insights, suggesting that *P. piptadeniae* may be regarded as a significant fungal pathogen within South American tropical forest biomes.

## RESULTS

### *Phellinotus piptadeniae* host and climate range

**Fig 1:**
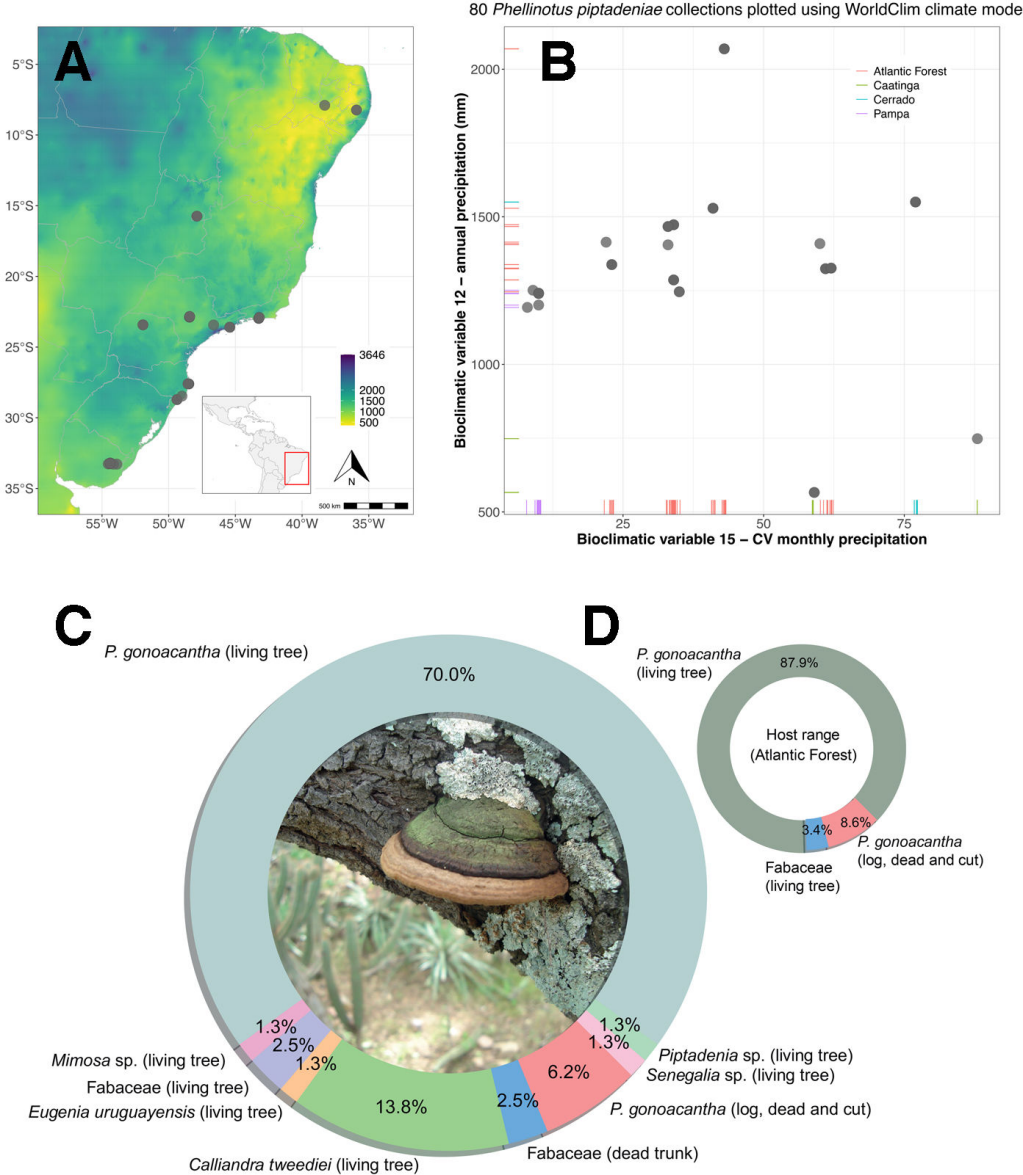
Geographical range of *Phellinotus piptadeniae* across a precipitation gradient (**A**) as retrieved from the WorldClim bioclimatic variable 12 (17). The species shows a higher ecological preference for wetter regions, mostly in the Atlantic Forest and Pampa phytogeographic domains, except for a few collections in the Caatinga (**B**). Total host range of *Phellinotus piptadeniae*, accounting for Brazil and Uruguay specimens (**C**) and across the Atlantic Forest only (**D**). Photo of *Phellinotus piptadeniae* in detail by E.R. Drechsler-Santos.

Across its full distribution range from northern Brazil to central Uruguay, *Phellinotus piptadeniae* has shown a higher ecological preference for the more humid regions of mostly the Atlantic Forest and Pampa phytogeographic domains (Fig. 1A and B). Annual precipitation in those regions ranges from around 1,200 to 1,500 mm (Fig. 1B). A few collection sites in the Caatinga seasonally dry woodlands of northeastern Brazil were also detected, where this fungal species grows in sites with less than 500 mm annual precipitation. All records in the savannas of the Cerrado and Pampa grasslands also came from relatively wet sites (Fig. 1B). *Phellinotus piptadeniae* basidiomata were predominantly found on *Piptadenia gonoacantha* living trees (Fabaceae). Fungal samples were also found growing on the legume *Calliandra tweediei* Benth. and *Eugenia uruguayensis* Cambess. (Myrtaceae) besides other unidentified species of the legume genera *Mimosa*, *Piptadenia*, and *Senegalia* (Fig. 1C and D; Table S1 [available at https://github.com/LBMCF/phepip_isotopes]).

### *Phellinotus piptadeniae* genome sequencing, assembly, and annotation metrics

Sequencing on the Illumina HiSeq 2500 platform generated 28,672,142 paired-end short reads, corresponding to 3,784,150,188 bases. Through sequencing on the Oxford Nanopore Technologies’ MinION, we obtained 409,847 long reads, corresponding to 772,618,969 bases. Using the MaSuRCA-Purge_dups assembly pipeline, we obtained an assembly with 418 contigs, 32,966,471 bp in length, in which the largest contig was 813,870 bp long, N50 of 203,956 bp, L50 of 45, and GC content of 47.73%.

The genome of *P. piptadeniae* has 9,771 protein-coding genes. Sequencing depths of 114× and 23× were obtained on the HiSeq 2500 and MinION platforms, respectively. The newly assembled genome possesses 95.6% of the orthologous genes searched through the BUSCO analysis. Among those orthologous genes, 93.3% were classified as single copy, 2.3% as duplicated, 1% as fragmented, and 3.4% as missing (see Fig. S1 at https://github.com/LBMCF/phepip_isotopes).

### Comparative genomics and phylogenomics of Hymenochaetales

**Fig 2:**
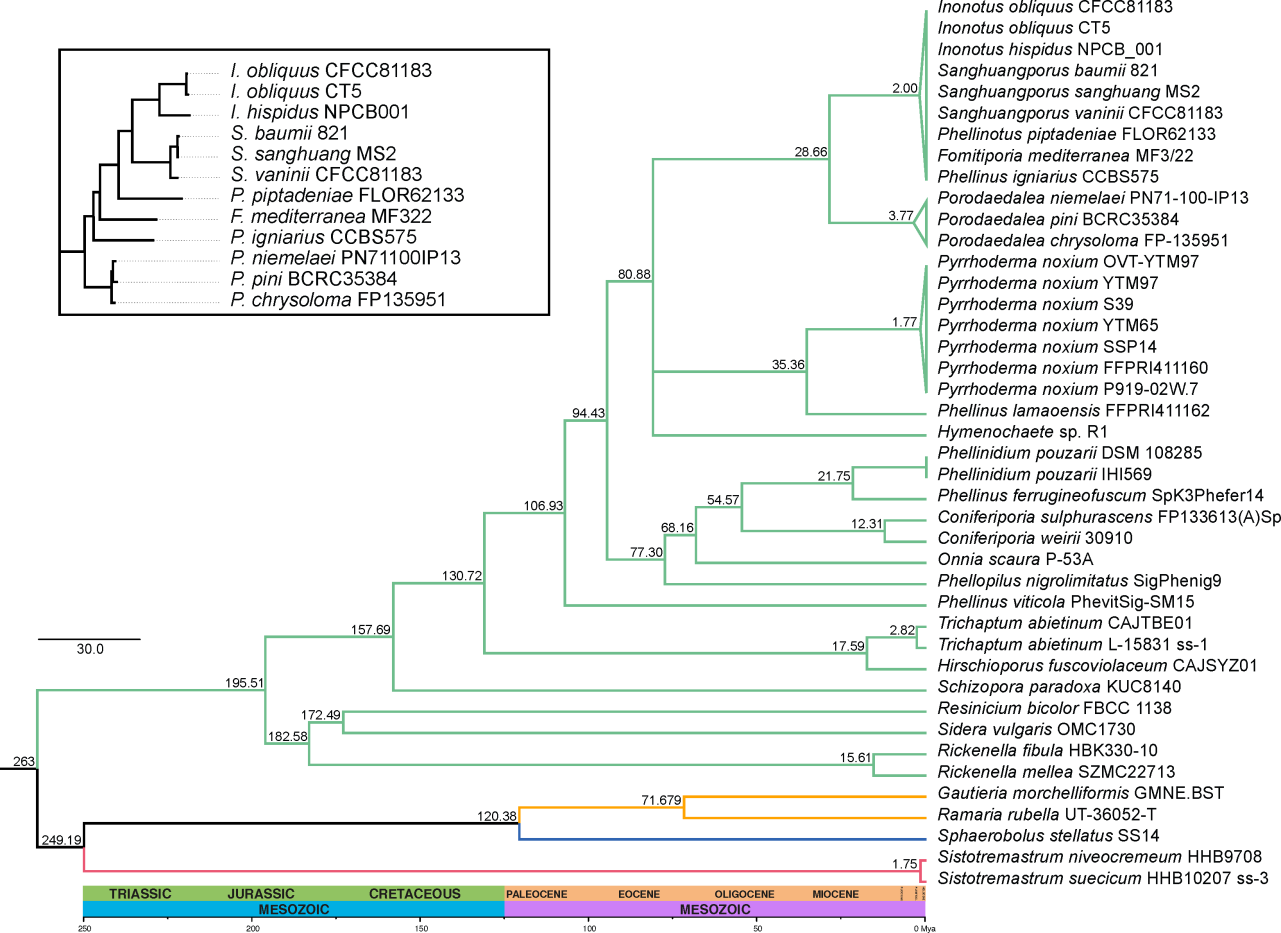
Phylogenomic dated tree showing the placement of *Phellinotus piptadeniae* in the context of the lineages *Hymenochaetales* (light green branches), *Gomphales* (orange), *Trechisporales* (magenta), and *Geastrales* (blue). The tree was calibrated using the MRCA of *Hymenochaetales* and *Trechisporales* (243 Mya), and the MRCA of *Phellinus igniarius* and *Fomitiporia mediterranea* (2.003 Mya). Other nodes were calculated using the least-squares method of IQTree. Clade separation containing *P. piptadeniae*, without time estimates, is shown in the detail box.

The dated phylogenomic tree (Fig. 2) suggests that *P. piptadeniae* diverged from its MRCA around 2 million years ago (Mya), in the Pleistocene. Our dating analysis could not obtain enough resolution to separate the clade containing *P. piptadeniae*, *Sanghuangporus*, *Inonotus*, *F. mediterranea*, and *P. igniarius* (Fig. 2). Nonetheless, before the dating analysis, we described that *P. piptadeniae* shares its MRCA with the genera *Sanghuangporus* and *Inonotus*, in a clade that diverged from *F. mediterranea* and *P. igniarius* (Fig. 2, detail box). All clades have a branch support of 100% (see Fig. S2 at https://github.com/LBMCF/phepip_isotopes), except for the clade including *P. noxium* FFPRI411160 and *P. noxium* P91902W7, with a support of 96%. The full list of genomes with respective accession codes is available in Table S2 (available at https://github.com/LBMCF/phepip_isotopes).

**Fig 3:**
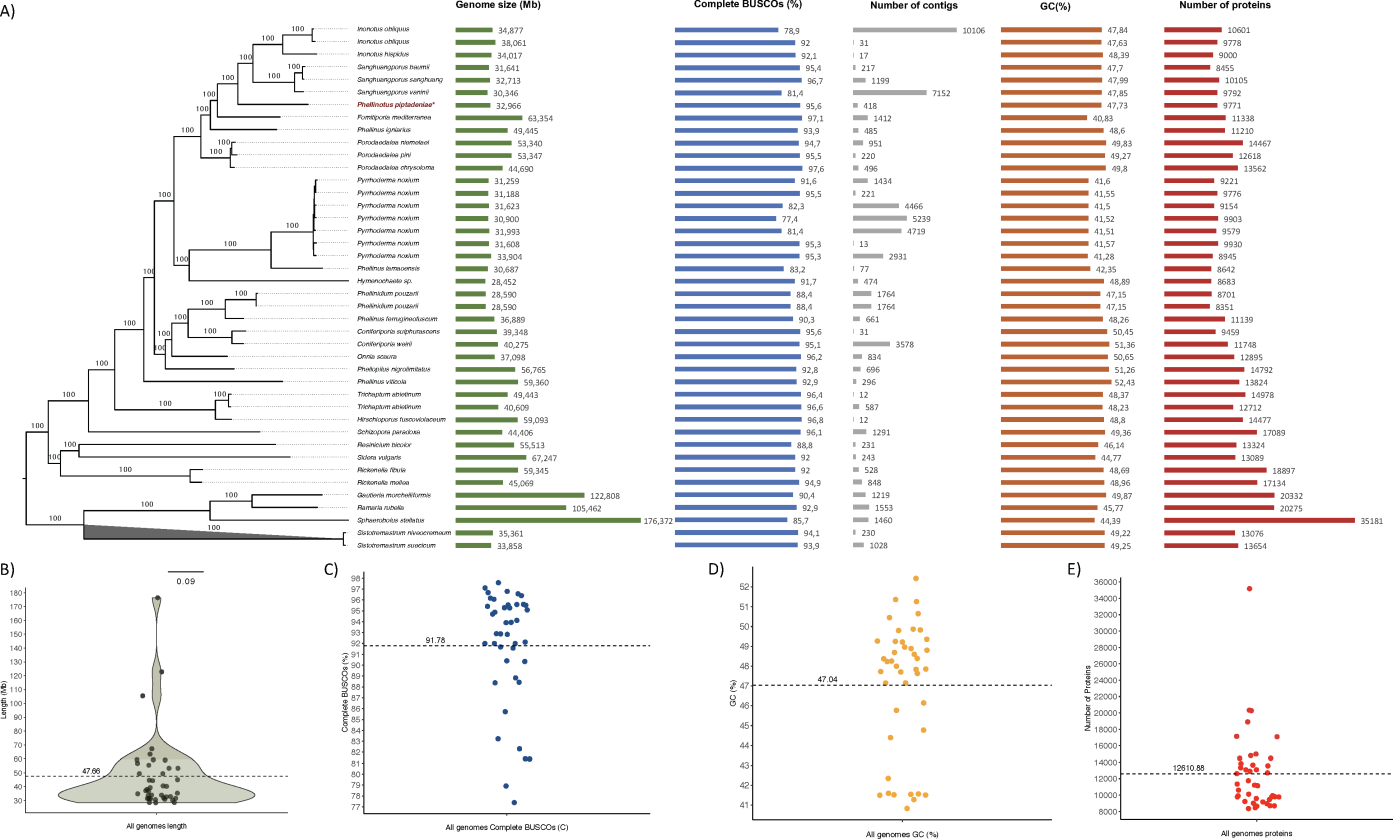
(**A**) Hymenochaetales maximum likelihood (ML) phylogeny plotted with data on genome size, gene completeness (BUSCO analysis), number of contigs, GC content, and number of proteins, including the genome of *Phellinotus piptadeniae*, 36 genomes belonging to other Hymenochaetales species, and five genomes belonging to the outgroup. (**B**) Dot and violin plots show genome size variation for Hymenochaetales and highlight the mean for the analyzed metric. (**C**) The dot plot shows genome completeness inferred using BUSCO. (**D**) The dot plot shows the variation of GC content for Hymenochaetales and highlights the average for the analyzed metric. (**E**) The dot plot shows the variation of predicted protein content for Hymenochaetales and highlights the average for the analyzed metric.

The genome size in Hymenochaetales (i.e., excluding the outgroup) ranged from 28.45 to 67.25 Mbp (mean = 41.30 Mbp, median = 37.10 Mbp) (Fig. 3A and B). The genome of *P. piptadeniae* is within the range expected for the order, and phylogenetically in a clade that contains species with similar genome sizes. The same is observed for the GC content, which ranged from 40.83% to 52.43% (mean = 46.95%, median = 47.99%), showing that the genome of *P. piptadeniae* has GC content close to both the expected mean and median, and the species is phylogenetically grouped with genomes with similar GC content (Fig. 3A through C). We also evaluated the completeness of the genome used in phylogenetic analysis (obtained from JGI and NCBI) for single-copy orthologous gene content. The completeness of genomes belonging to Hymenochaetales ranged from 77.4% to 97.6% (mean = 91.84%, median = 93.9%) (Fig. 3A through D). And, again, the genome of *P. piptadeniae* exhibits completeness greater than both mean and median for Hymenochaetales, demonstrating the quality of the genome generated in our study.

**Fig 4:**
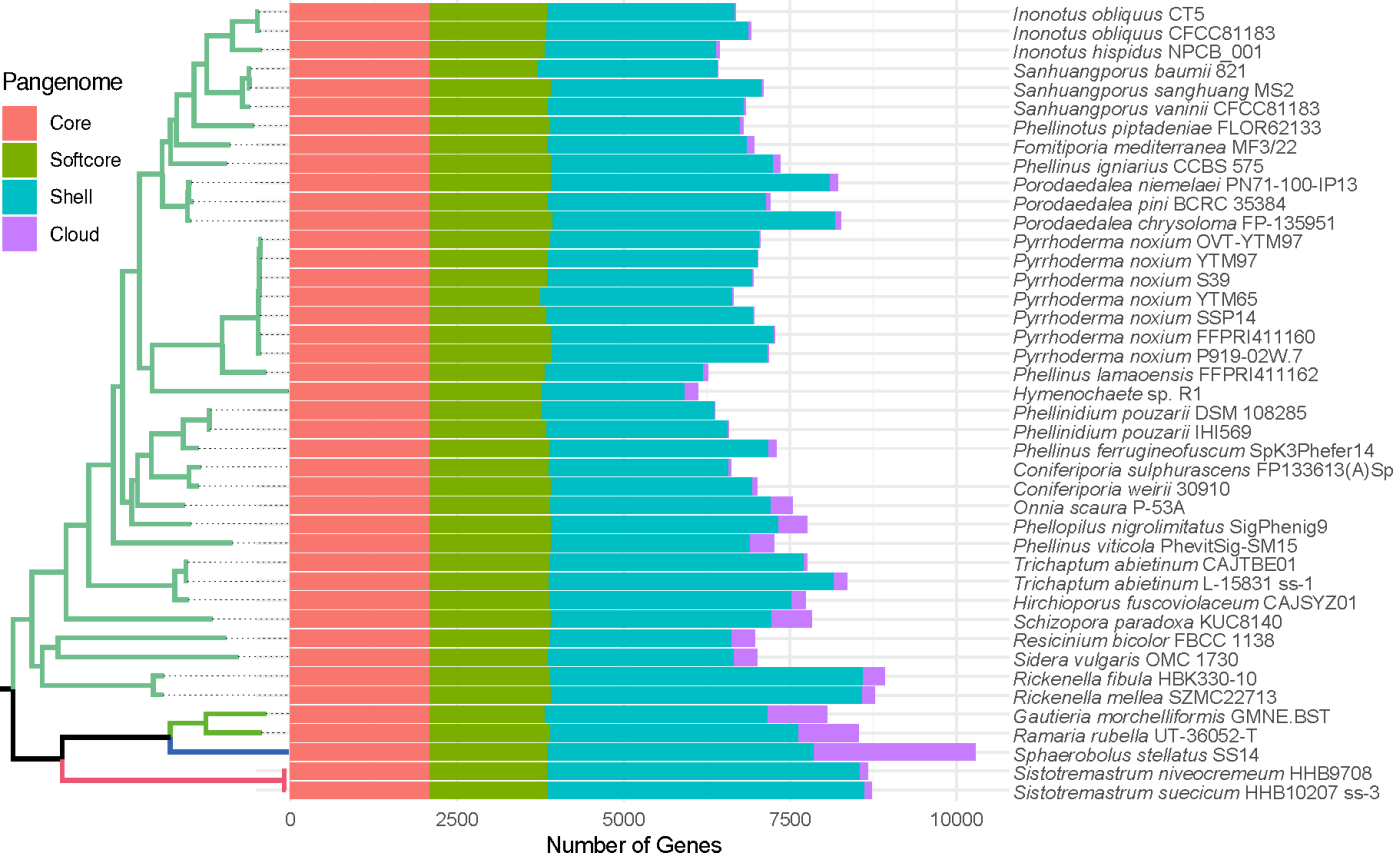
Pangenome distribution determined by OrthoFinder. Pangenome classes are as determined in Petersen et al. (18).

We obtained a pangenome of the selected species through ortholog family determination with OrthoFinder (Fig. 4). Orthogroup determination was performed as seen in Petersen et al. (18). In this ortholog distribution, a total of 27,834 orthogroups were obtained, out of which 2,099 orthogroups were designated as the core pangenome (present in all assemblies) and 1,834 orthogroups are part of the softcore pangenome (≥95% of the genomes). The variable part of the pangenome was composed of the shell (<95%, >2% of the assemblies), composed of 14,924 orthogroups, and the cloud pangenome (genome-exclusive orthogroups), composed of 8,977 orthogroups spread throughout assemblies. A full table with the numbers used in Fig. 4 is available in Table S3 (available at https://github.com/LBMCF/phepip_isotopes).

Regarding its genome structure, *P. piptadeniae* fits the average genome length and GC content. Relevant gene deletions could not be detected in this species either, in which 51 ortholog families were detected as species-exclusive (cloud pangenome, Fig. 4; Tables S3 and S4 [available at https://github.com/LBMCF/phepip_isotopes]). Over 70% of these species-exclusive ortholog families have disordered regions (36). In half of these families (18), all orthologs have been categorized as effectors, plus another 17 in which most or some orthologs were detected as effectors. Protein domains could only be identified in nine species-exclusive ortholog families. These include Hsp70 chaperones, membrane transporters, secondary metabolite and siderophore biosynthesis, DNA repair, proteolysis, and detoxification.

### Stable isotope and CAZy clustering analyses reveal a flexible lifestyle in *P. piptadeniae*

**Fig 5:**
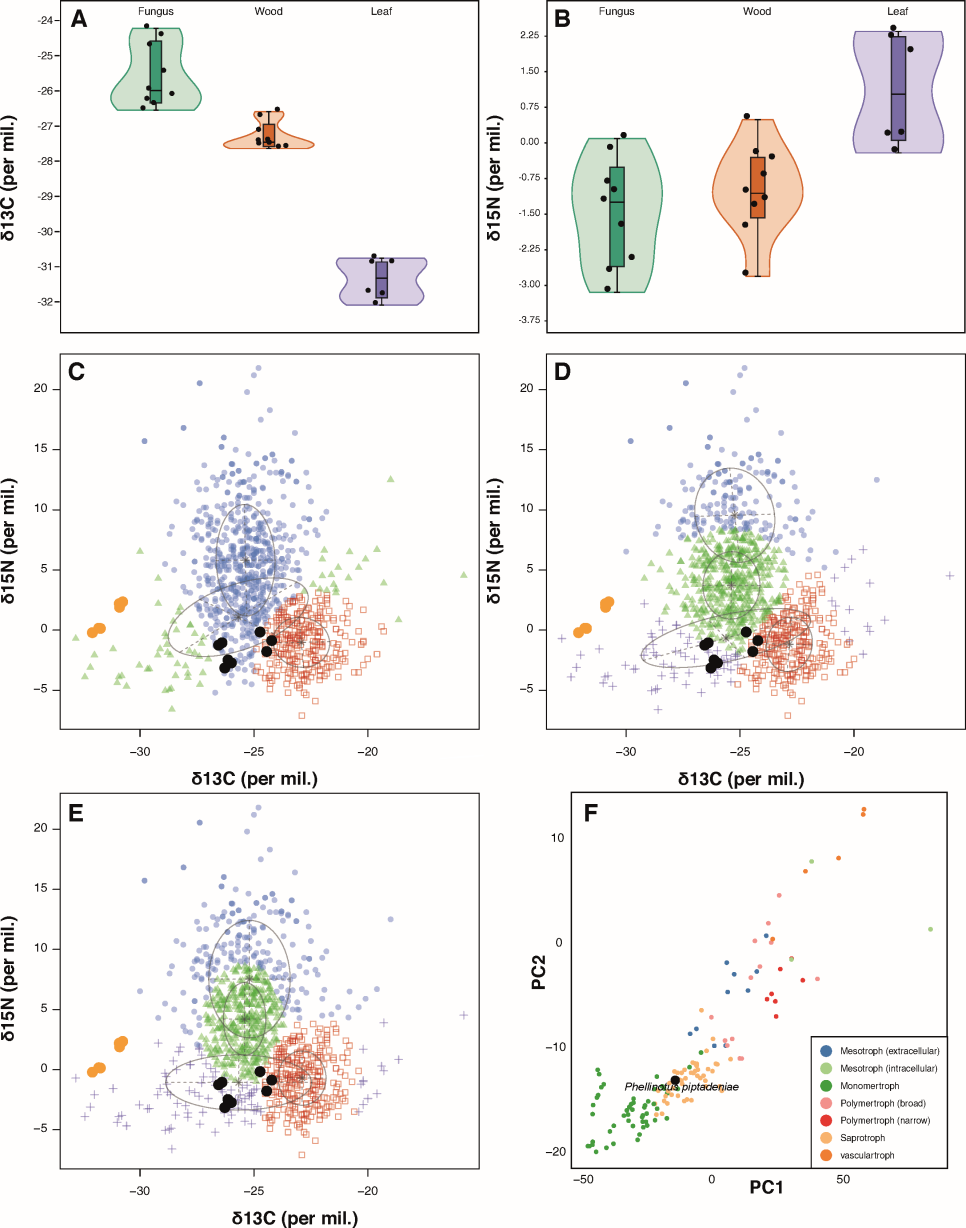
(**A-B**) δ^13^C and δ^15^N stable isotope patterns in *P. piptadeniae* basidiomata (fungus), *P. gonoachanta* branches (wood), and leaves (leaf) samples. (**A**) Values of δ^13^C (per mil.). (**B**) Values of δ^15^N (per mil.). (**C-E**) *Mclust* analysis of *P. gonoacantha* leaves (orange) and *P. piptadeniae* (black) samples from this study and 957 other samples compiled by Korotkin et al. (9). (**C**) *Mclust* with the VVV model and three components. (**D**) *Mclust* with the VEV model and four components. (**E**) *Mclust* with the VVI model and four components. (**F**) Catastrophy clustering of the CAZy composition of 99 fungal species compiled by Zhao et al. (4) plus *P. piptadeniae* (highlighted). Full cluster descriptions are available in Tables S5 and S6 (Fig. 5A through E), Fig. S3; Table S7.

A total of nine *Phellinotus piptadeniae* basidiomata were analyzed for δ^13^C and δ^15^N stable isotopes, deriving from distinct dead branches of living *Piptadenia gonoachanta* trees jointly with their leaves (Fig. 5A and B). The dead branches of the living trees were still completely attached to the trunk or detached from the trunk, but not on the soil. As expected, the leaves exhibited a significantly lower δ^13^C (*P* < 0.001, Fig. 5A) and a significantly higher δ^15^N (*P* < 0.001) than both wood from dead branches and fungal basidiomata (Fig. 5B). Regardless of sample origin (attached or detached from tree trunks), both δ^13^C (Fig. 5A) and δ^15^N (Fig. 5B) patterns were very similar, in which values were situated between tree leaves and fungal basidiomata values. Although the δ^15^N of fungal basidiomata and wood samples were quite similar (and thus not statistically different) (Fig. 5B), their δ^13^C values were distinct, with fungal basidiomata exhibiting higher and statistically significant (*P* < 0.001) values than tree wood (Fig. 5A).

In their study, Korotkin et al. (9) applied three *Mclust* models to separate isotope data from fungal samples; VVI (diagonal, varying volume, and shape), VEV (ellipsoidal, equal shape), and VVV (ellipsoidal, varying volume, shape, and orientation). The fitting model presented in Fig. 5A (VVV model with three components) appears to be too simplistic to reflect the niche diversity in the samples, while the models presented in Fig. 5B and C achieved a better resolution.

*Mclust* with the VVV model and three components. Cluster 1 (circles) contains 7/9 *P*. *piptadeniae* samples. Cluster 2 (squares) includes 2/9 *P*. *piptadeniae* samples. Cluster 3 (triangles) contains all leaf samples. (D) *Mclust* with the VEV model and four components. Cluster 1 (circles) does not contain any samples from this study. Cluster 2 (squares) includes 2/9 *P*. *piptadeniae* samples. Cluster 3 (triangles) 3/9 *P*. *piptadeniae* samples. Cluster 4 (crosses) contains all leaf samples and 4/9 *P*. *piptadeniae* samples. (E) *Mclust* with the VVI model and 4 components. Cluster 1 (circles) does not contain any samples from this study. Cluster 2 (squares) includes 2/9 *P*. *piptadeniae* samples. Cluster 3 (triangles) 2/9 *P*. *piptadeniae* samples. Cluster 4 (crosses) contains all leaf samples and 5/9 *P*. *piptadeniae* samples. (F) Catastrophy clustering of the CAZy composition of 99 fungal species compiled by Zhao et al. (4) plus *P. piptadeniae* (highlighted). Full cluster descriptions are available in Tables S5 and S6 (Fig. 5A through E), Fig. S3; Table S7 (Fig. 5F) (all available at https://github.com/LBMCF/phepip_isotopes).

The clustering pattern for the *P. piptadeniae* samples is practically identical in Fig. 5B and C, with just one sample belonging to Cluster 3 with the VEV model and switching to Cluster 4 with the VVI model. Taking VVI as an example of the *P. piptadeniae* samples clustering, our nine fungal samples were divided into Cluster 2 (squares—two samples) composed of 76.49% saprotrophic fungi; Cluster 3 (triangles—two samples) composed of 62.48% ectomycorrhizal samples and 25.33% bryophilous Hymenochaetales; and Cluster 4 (crosses—5 samples) composed of 40% bryophilous Hymenochaetales, 9.27% saprotrophic, and 5.9% ectomycorrhizal fungi (Table S5 [available at https://github.com/LBMCF/phepip_isotopes]). Kruskal-Wallis results and subsequent Dunn test strongly support δ^15^N and δ^13^C stable isotope mean differences between each of the recovered clusters (Table 1). Subsequently, we tested whether *P. piptadeniae* in cluster 1 (best-fit model) has stable isotope values similar to those of known saprotrophic, ECM (ectomycorrhizal), bryophilous Hymenochaetales, NS-NE (neither saprotrophic nor ectomycorrhizal), and AUTO (autotrophic) taxa (Table 2). The latter category includes the moss species *Dicranum scoparium*, a control for the original analysis, and leaf samples from *Piptadenia gonoacantha,* as a control for our analysis.

**TABLE 1:** Comparison of trophic cluster assignments by *Mclust* to test differences in the sampling utilizing Kruskal-Wallis and Dunn tests^*a*^

Test	Comparison	δ^15^N	δ^13^C
Kruskal-Wallis	All clusters	Chi-square = 719.12 df = 3 *P* < 0.00001	Chi-square = 480.24 df = 3 *P* < 0.00001
Dunn	Cluster 1 vs cluster 3	*P* < 0.0001	*P* < 0.0001
	Cluster 1 vs cluster 2	*P* < 0.0001	*P* < 0.0001
	Cluster 2 vs cluster 3	*P* < 0.0001	*P* < 0.0001

^*a*^ Stable isotope data from the compilation of Korotkin et al*.* (9) and data generated herein for *P*. *piptadeniae* (Table S6).

**TABLE 2:** Comparison of stable isotope results to test statistical differences between trophic clusters and trophic states using Kruskal-Wallis and Dunn tests^*a*^

Test	Trophic comparison	δ^15^N	δ^13^C
Kruskal-Wallis	*P. piptadeniae*; ECM; SAP; NS-NE; “unknown”	Chi-square = 404.18 df = 5 *P* < 0.00001	Chi-square = 464.13 df = 5 *P* < 0.00001
Dunn	*P. piptadeniae vs* AUTO	*P* = 0.2145	*P* = 0.0059
	*P. piptadeniae* vs ECM	*P* < 0.0001	*P* = 0.3575
	*P. piptadeniae* vs NS-NE	*P* < 0.0001	*P* = 0.3495
	*P. piptadeniae* vs SAP	*P* = 0.1717	*P* < 0.0001
	*P. piptadeniae* vs “Unknown”	*P* = 0.0918	*P* = 0.3903

^*a*^ ECM = ectomycorrhizal; NS-NE = neither saprotrophic nor ectomycorrhizal (the other biotrophic ones); SAP = saprotrophic; AUTO = Autotrophic. Stable isotope data from the compilation of Korotkin et al*.* (9) and data generated herein for *P*. *piptadeniae* (Table S5).

**Fig 6:**
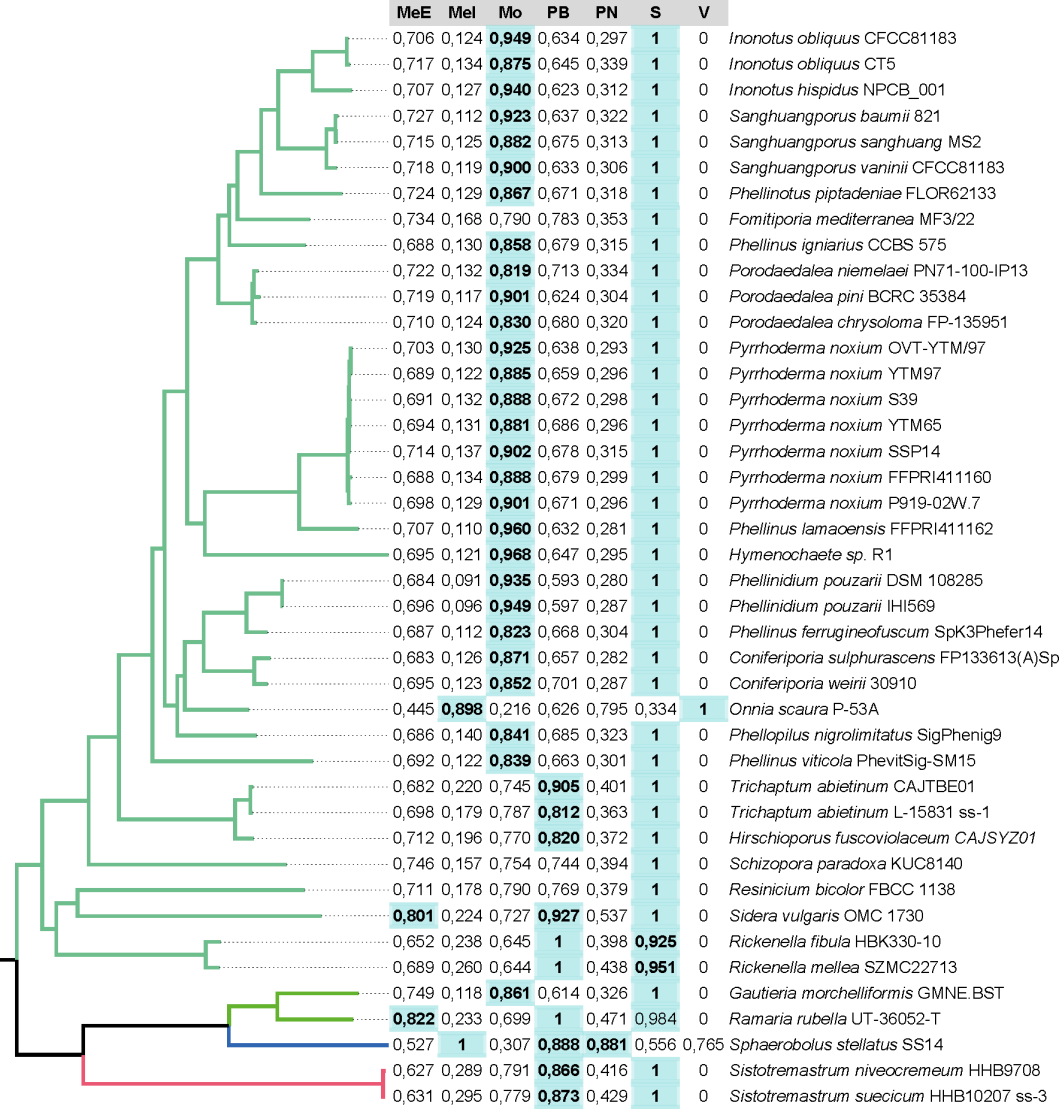
Trophic mode scores based on the CAZy profile. Scores higher than 0.8 are highlighted in bold and green background. MeE = Extracellular mesotrophs, MeI = Intracelullar mesotrophs, Mo = Monomertroph, PB = Broad host-range polymertrophs, PN = Narrow host-range polymertrophs, S = Saprotroph, and V = Vasculartrop.

A CAZy-based classification predicted *P. piptadeniae*, as well as all other Hymenochaetales and most other neighboring clades as saprotrophs (Fig. 4D and 6). Nonetheless, as explained by the authors, all trophic modes with a score higher than 0.8 are relevant, which then classifies *P. piptadeniae* and many neighboring genomes as possible Monomertrophs, which Hane et al. (19) describe as either symbionts or biotrophs. Notable exceptions are *F. mediterranea*, with a Monomertroph score of 0.790, and *Onnia scaura*, which presented an outlier profile and, thus, should not be considered.

## DISCUSSION

The combination of genomics, stable isotope analyses, host tree association, and bioclimatic data has revealed more details of the interaction of *Phellinotus piptadeniae* and its main host, *Piptadenia gonoacantha* (Fig. 1C and D). Overall, we verified that the trophic mode of *P. piptadeniae* involves traits of biotrophy added to its previously known saprotrophic lifestyle. This could be a determinant of its host specificity and interaction with plant hosts.

The entire genus *Phellinotus* predominately occurs in the South American seasonally dry tropical forest (SDTF) biome (20), where each species often occurs associated with disjunct SDTF areas (13, 14). While also occurring in drier settings, *P. piptadeniae* seems to have deviated from the ancestral ecological niche of the genus to thrive in more humid settings of the Atlantic Forest, Pampa, and Cerrado phytogeographic domains (Fig. 1A and B). Whether the successful ecological adaptation of *P. piptadeniae* into these settings was enabled by the evolution of its flexible lifestyle, involving both the saprotrophy and the herein-described pathotrophic specialization, remains an open question.

### Insights into the trophic mode of *Phellinotus piptadeniae* as revealed by phylogenomics

Our phylogenomic analysis placed *Phellinotus piptadeniae* in a strongly supported clade of closely related genera and species including *Sanghuanporus*, *Inonotus,* and *Phellinus igniarius*, all of which are wood-decay fungi with medicinal applications (21, 22), and *Fomitiporia mediterranea*, a broad-range trunk pathogen affecting grapevines (23), citrus (24), and olives (25) (Fig. 2 and 3). Although assembly and annotation metrics fit the variability found in Hymenochaetales (Fig. 3), *P. piptadeniae* differs from the other species for its narrow host specificity, considering its preference for *P. gonoacantha* and its limitation to other legume trees. Even though a close relationship of *P. piptadeniae* with those hymenomycetous genera was previously suggested based on a few genomic regions (15), their strong relatedness and divergence date based on genomic-wide data had not been estimated before. Our analysis obtained a single MRCA for these species around 2 Mya (Fig. 2).

This study presents new information about the origins of *P. piptadeniae*; however, we highlight the large gap of complete genomes available for this analysis. A previous phylogenetic study based on a concatenated set of genomic regions (15) mentioned several other genera (e.g., *Fomitiporella*, *Inocutis*, and *Fulviformes*) as closer to *P. piptadeniae* than *Sanghuangporus*. Unfortunately, none of those genera have assembled genomes available in public databases. The addition of such genomes could provide a better resolution in understanding which genes are in fact part of the “cloud” genome of *P*. piptadeniae (Fig. 4; Table S4 [available at https://github.com/LBMCF/phepip_isotopes]). The closeness of *P. piptadeniae* to species on the edge of saprotrophy and pathogenic lifestyles lays the basis for our working hypothesis. While *P. piptadeniae* is strongly associated with living *P. gonoacantha* trees with no apparent pathogenic symptoms, could it be associated with a biotrophic lifestyle? Altogether, our results indicate that this is possible.

Several Hymenochaetaceae macrofungi are known to be tree pathogens, such as *Porodeaedalea pini* (Brot.) Murrill and *Phellinus tremulae* (Bondartsev) Bondartsev & P.N. Borisov, including species phylogenetically related to *P. piptadeniae* [such as *Fulvifomes fastuosus* (Lév.) Bondartseva & S. Herrera and *Tropicoporus linteus* (Berk. & M.A. Curtis) L.W. Zhou & Y.C. Dai] and a congeneric species [*Phellinotus badius* (Cooke) Salvador-Mont., Popoff & Drechsler-Santos] (11). These species, however, are usually pointed out to degrade the heartwood (dead organic matter within living trees), which could be a reason to classify them as saprotrophic. The work by (26) reports this saprotrophic and biotrophic lifestyle plasticity in other wood-decay basidiomycetes, which could lay the ground for understanding the trophic mode for *P. piptadeniae* and related species. Nonetheless, the aforementioned study concentrates on the plasticity of ectomycorrhizal species, which are generally contained in different clusters from *P. piptadeniae* in our stable isotope analysis, showing an opportunity for further studies in this matter.

### Stable isotope analysis and CAZy content jointly suggest a pathotrophic lifestyle in *P. piptadeniae*

Conversely expected, considering the lignicolous habit of *P. piptadeniae* and previous results regarding the trophic modes of lignicolous fungi based on data from stable isotopes (9), our clustering analysis based on stable isotope data of *P. piptadeniae* and other species placed it outside the cluster that includes the vast majority of saprotrophic fungi (Fig. 5C and E; Tables S5 and S6 [available at https://github.com/LBMCF/phepip_isotopes]). Bioinformatics analyses based on the content of enzymes related to C metabolism also indicate a dichotomy in its feeding strategy, as well as other species in the order (Fig. 5F and 6; Table S7 [see at https://github.com/LBMCF/phepip_isotopes]).

Although the approach of radiocarbon isotopes (27) and stable isotopes have been mainly used for separating mycorrhizal fungi from saprotrophs (7), different outcomes have also been reported. For instance, the assemblage retrieved by Korotkin et al. (9) includes clusters containing a miscellaneous assemblage without a clear dominance of any specific trophic mode (including saprotrophs, ectomycorrhizal, and putative parasites or endophytes), which is where the majority of *P. piptadeniae* samples are located, when we added our data to their compilation (Fig. 5C through E). Furthermore, *P. piptadeniae* samples in this cluster share similar C ratio signatures as ECM, NS-NE, and bryophilous Hymenochaetales groups. On the other hand, their N ratio signatures are significantly different compared to ECM and NS-NE taxa but similar to saprotrophs and bryophilous Hymenochaetales. Korotkin et al. (9) observed a similar pattern for some bryophilous Hymenochaetales, speculating that they might obtain their carbon directly from their photosynthetic host. A recent and extensive study on different species of the wood saprothrophic genus *Mycena* (Basidiomycota, Agaricales) in distinct biomes and with several tree species indicated that *Mycena* spp. are also opportunist-generalist plant root invaders (28). Moreover, the aforementioned authors indeed emphasized that the conventional and widely used strict separation of macrofungi into the rigid ecological categories of mutualist, parasite/pathogen, or saprotroph has been frequently questioned, as reported in a study encompassing data from microcosm tests with 201 species of wood-decay searching for facultative biotrophy in saprotrophic basidiomycotan fungi (26). Although this evidence (Fig. 5 and 6,) points toward a non-saprotrophic trophic mode for *P. piptadeniae*, it is worth noting that distinct samples of the same species may group in different clusters in the stable isotope approach, putatively indicating distinct trophic modes. Nonetheless, this might be due to plasticity of carbon sources within that species (e.g., different samples with different habits), as previously investigated for distinct basidiomycotan saprothrophic macrofungi using combined carbon and nitrogen concentrations, isotopic ratios (^13^C:^12^C, ^15^N:^14^N, and ^14^C:^12^C), and compositional patterns in wood, cellulose, and basidiomata (29).

Based on field observation of wood decomposition, it has been previously known that some Hymenochaetaceae plant pathogens are capable of degrading the sapwood from living trees (2, 11). Furthermore, *Ganoderma sessile* (Ganodermataceae, Polyporales) had already been experimentally inoculated in young trees (which possess only sapwood) of pine and oak and, around 1 year later, reisolated outside of the inoculation point, indicating the capacity to infect living sapwood (30). *Phellinotus piptadeniae* fruiting bodies are commonly found on high branches of *Piptadenia gonoacantha* but rarely on the main trunk (Fig. 1C and D). Those branches likely exhibit poorly differentiated heartwood. Therefore, we hypothesize that *P. piptadeniae* might infect the living sapwood of *P. gonoacantha*, acquiring carbon from the living part of its host, explaining the pattern observed in our combined genomic stable isotope analysis.

## MATERIAL AND METHODS

### *Phellinotus piptadeniae* basidiomata occurrence and host association

The occurrence of the fungal species *Phellinotus piptadeniae* on living and dead host plants, its range and geographic distribution were obtained and confirmed from previously published systematic and biogeographic studies (14, 16), as well as from online databases (GBIF, https://www.gbif.org/; speciesLink, https://specieslink.net/; and MyCoPortal, https://www.mycoportal.org/portal/). A total of 80 taxonomically verified georeferenced herbarium collections of *P. piptadeniae* were assembled from the FLOR, IAC, HUEM, URM, and MVHC herbaria (Table S1 [available at https://github.com/LBMCF/phepip_isotopes]).

From the latitude and longitude of each collection, we extracted the monthly precipitation, and minimum and maximum temperatures using the WorldClim v.2.0 model layers (17) and the R package *raster* (31). The bioclimatic variables BIO12 (Annual Precipitation) and BIO15 (Coefficient of Variation of the Precipitation Seasonality) were derived from these climate models using the *extract* function of *raster*. We mapped the entire range distribution of *Phellinotus piptadeniae* against the BIO12 bioclimatic variable, using the R packages *raster*, *ggplot2* (32), *ggspatial* (33), and *rnaturalearth* (34). The bioclimatic space of *P. piptadeniae* was also assessed with a scatterplot that shows its distribution in different Brazilian phytogeographic domains and across the BIO12 and BIO15 axes.

For morphological and downstream genomic analyses, dehydrated basidiomata from a newly collected specimen of *P. piptadeniae* was obtained from a living tree of *Piptadenia gonoacantha* in the Atlantic Rainforest of southern Brazil (Parque do Córrego Grande, Florianópolis, State of Santa Catarina; Latitude: 27.599814 W, Longitude: 48.511375 S) and deposited at the FLOR fungarium under the voucher number FLOR62133. Macromorphological and micromorphological studies of collected basidiomata were made for fungal identification. For mycelium isolation, a piece from the collected fruiting body was removed and had its surface disinfected using 70% alcohol and distilled water, and subsequently inoculated in a 90 mm Petri dish containing MEA culture medium (2% malt extract, 2% glucose, and 2% agarose) supplemented with chloramphenicol. The Petri dish was incubated at 28 ± 2°C for 10 days and the fungal growth was daily checked.

### Genomic DNA extraction and sequencing

*Phellinotus piptadeniae* CCMB738 was inoculated in MEA culture medium and incubated at 28 ± 2°C for 10 days. After growth, the mycelium was removed from the Petri dish. The total DNA was extracted using the FastDNA Spin Kit (MP Biomedicals, Irvine, California, USA) for sequencing on the HiSeq 2500 platform (Illumina, San Diego, California, USA) and the ZymoBIOMICS DNA Miniprep Kit (Zymo Research, Irvine, California, USA) for sequencing on the MinION platform (Oxford Nanopore Technologies, Oxford, UK). DNA was evaluated qualitatively in 1% agarose gel and quantitatively by Nanodrop 1000 ND spectrophotometer (Thermo Scientific, Waltham, Massachussetts, USA) and Qubit fluorometer (Invitrogen, Waltham, Massachussetts, USA). Paired-end sequencing on the HiSeq 2500 platform was carried out from 1 µg of DNA, using the NEBNext Fast DNA Fragmentation and Library Preparation Kit (New England Biolabs, Ipswich, Massachusetts, USA). For MinION sequencing, genomic DNA was fragmented to 8 Kbp using the Covaris g-TUBE (Covaris, Woburn, Massachussetts, USA) and purified using AMPureXP beads reagent (Beckman Coulter Inc., Brea, California, USA). Subsequently, the library was prepared following the protocol described by Tomé et al. (35) and sequenced for 24 hours using the MinKNOW software with real-time base calling.

### Raw data processing, *de novo* genome assembly, and annotation

Raw reads from the Illumina platform were evaluated for quality using FastQC v0.11.5 software (36). Subsequently, the bases with a Phred score equal to or less than 20 were trimmed using the BBDuk software (37). The raw reads obtained through MinION sequencing were demultiplexed and had the adapters trimmed using the Porechop software (38). The *P. piptadeniae* genome was assembled using the *de novo* approach and the MaSuRCA-Purge_dups hybrid assembly pipeline, described by Tomé et al. (35). This assembly workflow uses the MaSuRCA software to generate a primary assembly from both short-reads (HiSeq 2500) and long-reads (MinION), and the Purge_Dups program to identify and remove haplotypic duplications (39, 40). The generated genome was evaluated for length, largest contig size, N50, and L50 metrics, using QUAST v4.6.0 software (41). The BUSCO v4 software (Benchmarking Universal Single-Copy Orthologs) was used to verify the completeness of single-copy orthologous genes (42). For this analysis, the basidiomycota_odb10 database was used.

Genome annotation was carried out using the Funannotate pipeline v1.8.17 (43), using 114030 proteins and 12293 ESTs from Hymenochaetales as evidence. Briefly, transcript evidence (ESTs) was aligned to the genome using minimap2 (44), while protein sequences were aligned using Diamond/Exonerate (45, 46). Conserved orthologs were identified with BUSCO (42), and the gene prediction was performed using GeneMark-ES (47), Augustus (48), SNAP (49), Glimmer HMM (50), and EVidenceModeler (51). The tRNA genes were predicted using tRNAscan-SE (52).

### Prediction of carbohydrate-active enzymes

The carbohydrate-active enzymes (CAZy) were identified from the predicted proteins file, either from Funannotate pipeline v1.8.17 (43) or from GenBank and JGI using the Catastrophy software v0.1.0 (19). Catastrophy uses CAZy predictions from HMMER (53) to infer lifestyle in plant-infecting fungi.

### Comparative genomics, phylogenomics, and homology assignment

Homology assignment of gene families was performed using OrthoFinder v2.5.2 (54). The taxon sampling in this analysis comprises *P. piptadeniae* and 41 other species of Hymenochaetales, Trechisporales, Gomphales, and Geastrales (the species from the latter three orders as outgroup) for which public genomes were available (Table S2; available at https://github.com/LBMCF/phepip_isotopes). The species-level phylogenomic tree was inferred using 1,123 single-copy orthologous gene families whose proteins were aligned using the built-in MAFFT module in OrthoFinder with default parameters and then concatenated in a supermatrix. We used IQ-TREE v. 2.1.2 (55) for phylogenomic inference with the parameter “-m TEST” to calculate the best fitting model using BIC criteria, which was JTT + F + I + G4, with 1,000 ultra-fast bootstrap replicates for branch support. IQ-TREE was also used for the time calibration of the tree with the least-squares analysis and two calibration points obtained from the TimeTree database (56): 243 Mya for the most recent common ancestor (MRCA) of *Hymenochaetales* and *Trechisporales*, and 2.003 Mya for the MRCA of *Phellinus igniarius* (L.) Quél. and *Fomitiporia mediterranea* M. Fisch.

### Stable isotope analysis

The carbon and nitrogen isotope ratios were determined by combustion using an elemental analyzer (Carlo Erba, CHN-1100) coupled to a Thermo Finnigan Delta Plus mass spectrometer at the Laboratory of Isotope Ecology of the Centro de Energia Nuclear na Agricultura (CENA/Universidade de São Paulo), in Piracicaba, State of São Paulo, Brazil. Isotope ratios are reported in per mil (‰), where δ^13^C is reported relative to the Vienna Pee Dee Belemnite (VPDB; ^13^C:^12^C ratio = 0.01118) standard and δ^15^N is reported relative to atmospheric air (AIR; ^15^N:^14^N ratio = 0.0036765). Internal standards (sugarcane leaves) are routinely interspersed with target samples to correct for mass effects and instrumental drift during and between runs. Long-term analytical error for the internal standards was 0.2‰ for both δ^13^C and δ^15^N, 1% for organic C, and 0.02% for total N. The carbon and nitrogen isotope ratios of field-collected samples of *P. piptadeniae*, and leaf samples from *P. gonoacantha* were determined by combustion using an elemental analyzer coupled to a mass spectrometer.

The samples generated in this study were added to the compilation provided by Korotkin et al. (9) and their clustering analysis was repeated. Briefly, δ^13^C and δ^15^N values were clustered with the *Mclust* package in R (57). The three fitting models presented by Korotkin were replicated: VVV with three components, VEV, and VVI both with four components. The categories set by the authors were saprotrophic (SAP), ectomycorrhizal (ECM), neither saprotrophic nor ectomycorrhizal (NS-NE), bryophilous Hymenochaetales (their test data set), and *Dicranum scoparium* Hedw. samples as a positive control for autotrophs. We tested the data for normality (values of δ^13^C and δ^15^N) to decide the application of one-way ANOVA or the Kruskal-Wallis test (58) to test the difference between the groups recovered in the best-fit model of *Mclust* analysis. As the data did not exhibit a normal distribution, we applied the Kruskal-Wallis test following the application of Dunn’s test (59) for testing for differences between each pair of recovered groups. Similarly, we applied the same tests to test the null hypothesis of no differences between *P. piptadeniae* values of δ^13^C and δ^15^N and trophic groups (SAP, ECM, NS-NE, AUTO, and bryophilous Hymenochaetales) recovered in the same cluster (considering the best-fit model). Tests were performed in R with in-house functions and the *dunn.test* package (60).

## Data Availability

All Supplementary Tables and Figures are available at the GitHub repository of this study (https://github.com/LBMCF/phepip_isotopes).
